# The willingness to receive sexually transmitted infection services from public healthcare facilities among key populations at risk for human immunodeficiency virus infection in Bangladesh: A qualitative study

**DOI:** 10.1371/journal.pone.0221637

**Published:** 2019-09-04

**Authors:** Gorkey Gourab, Mohammad Niaz Morshed Khan, A. M. Rumayan Hasan, Golam Sarwar, Samira Dishti Irfan, Md. Masud Reza, Tarit Kumar Saha, Lima Rahman, A. K. M. Masud Rana, Sharful Islam Khan

**Affiliations:** 1 Programme for HIV and AIDS, International Centre for Diarrhoeal Diseases Research, Dhaka, Bangladesh; 2 Universal Health Coverage, Health System and Population Studies Division, International Centre for Diarrhoeal Diseases Research, Dhaka, Bangladesh; 3 Institute of Public Health (IPH), Dhaka, Bangladesh; 4 HIV/AIDS Program, Health, Nutrition and HIV/AIDS Sector, Save the Children, Dhaka, Bangladesh; University of Toronto, CANADA

## Abstract

**Background:**

In Bangladesh, community-based and peer-led prevention interventions for human immunodeficiency virus infection are provided to key populations (KPs) by drop-in centers (DICs), which are primarily supported by external donors. This intervention approach was adopted because public healthcare facilities were reportedly insensitive to the needs and culture of KPs, particularly with regard to the provision of sexually transmitted infection (STI) services. Nonetheless, in the absence of external funding, STI services need to be integrated into public healthcare systems.

**Methods:**

A qualitative study was conducted in 2017 to understand the willingness of KPs to uptake the STI services of public healthcare facilities. Data were collected based on 34 in-depth interviews, 11 focus group discussions, and 9 key informant interviews. The social-ecological theoretical framework was used to analyze the data thematically and contextually.

**Results:**

Most participants were either resistant or reluctant to uptake STI services from public healthcare facilities because of their previous firsthand experiences (e.g., disrespectful and judgmental attitudes and behaviors), perceived discrimination, anticipatory fear, and a lack of privacy. Very few participants who had visited these facilities to receive STI services were motivated to revisit them. Nevertheless, they emphasized their comfort in DICs over public healthcare facilities. Thus, it appears that KPs can be situated along a care-seeking continuum (i.e., resistance to complete willingness). Unless policymakers understand the context and reasons that underlie their movement along this continuum, it would be difficult to encourage KPs to access STI services from public healthcare facilities.

**Conclusion:**

KPs’ willingness to uptake the STI services of public healthcare facilities depends not only on individual and community experiences but also on the nexus between socio-structural factors and health inequalities. Community mobilization and training about the needs and culture of KPs for healthcare professionals are essential. Therefore, addressal of a wide range of structural factors is required to motivate KPs into seeking STI services from public healthcare facilities.

## Introduction

Human immunodeficiency virus (HIV) infection is still considered to be an alarming public health burden in low-and lower-middle income countries (LMIC). Indeed, it is ranked as the 17^th^ leading cause of death in South Asia [1, 2]. Despite the decreasing prevalence of HIV across the past decade, the associated disease burden is still alarming in some LMIC[1–3]. Bangladesh has witnessed a 10% increase in the incidence of HIV infections across the past decade[4]. This increase is especially prominent among one of the key populations (KPs) that is at risk for HIV infection. For example, the results from the last serological surveillance of 2016 that was conducted among people who inject drugs (PWID) in the city of Dhaka showed that 22% of the sampled male PWID and 5% of the sampled females who inject drugs(FWID) were HIV positive [5]. The HIV epidemic was particularly centered in one specific neighborhood in Dhaka city, where 27.3% of the sampled male PWID were HIV positive [5]. Therefore, there is a risk that HIV infection may transcend KPs and eventually be transmitted to the general population [6].

HIV prevention interventions that targeted KPs that are at risk for HIV, including female sex workers (FSW), men who have sex with men (MSM), transgender women (known as *hijra* in South Asia), and PWID, were introduced in the early 1990s. These interventions adopt a community-based and peer-led approach, whereby all the activities are operated through static service delivery points that are known as drop-in centers (DICs); prevention services are provided through these DICs [7–9]. This approach raises awareness about safer sex, condom and lubricant, and the perils of needle-syringe exchange (to PWID) through outreach activities that are offered during sex trades and at drug-taking and social gathering spots where KPs typically congregate during particular times. In addition, sexually transmitted infection (STI) management, counseling, HIV testing services (HTS), and recreational activities are provided through DICs, where all the services are provided free of cost [9–11]. The Joint United Nations Programme on HIV/AIDS (UNAIDS) considers the DIC-based approach to be the “best practice.” However, in the absence of support from donors, this approach utilizes limited resources to combat the epidemic [12–14]. Moreover, the DIC-based approach, which is funded by donors and operated by non-governmental organizations (NGOs), has emerged as structures that are parallel to national health systems, thereby leading to inefficiencies in many countries [15–17].

Sustainable service delivery through DICs is one of the major challenges in the national HIV response due to donor dependency. Hence, “sustainable strategies must be built into project design and implementation to enable HIV efforts to continue long after donor-supported projects are completed” [18]. To build resilient and sustainable health systems, several countries have opted for an integrated model to explore the possibility of safeguarding the national HIV response in the absence of donations. Some of these approaches include stigma-reducing initiatives for MSM in Kenya and the Avahan intervention in India, which has successfully transitioned into a phased program in government healthcare facilities [18–22].

Several factors, such as stigma, discrimination, and criminalization deter access to healthcare. Beyrer et al. (1999) claimed that “for people who sell sex (SW) or inject drugs (IDU) and for men who have sex with men (MSM), stigma, discrimination, and criminalization can limit access to care, inhibit service uptake, and reduce the disclosure of risks” [23]. Similarly, in Bangladesh, KPs are less willing to uptake healthcare facilities because the aforementioned factors have a more pronounced effect on them due to their identities or behaviors, which are prohibited by the law. For example, same-sex sexual behaviors and illicit drug use are criminalized by the Bangladesh Penal Code (Section 377) and the Narcotics Control Act (1990), respectively. In addition, several laws in Bangladesh criminalize sex work (e.g., Human Trafficking (Deterrence and Suppression) Act 2012; Section 373, Penal Code, 1860)[24, 25]. Several studies that have been conducted in Bangladesh have shown that public healthcare facilities are not ready to serve the needs of KPs in culturally sensitive ways [26–28]. These studies have underscored the following barriers that deter KPs from uptaking healthcare services: fear of HIV infection among physicians who work at public healthcare facilities, limited access to healthcare services among *hijra*, and the reluctance of KPs to disclose their sexual behaviors to physicians. Specifically, Ullah et al. (2011) found that physicians’ anticipatory fear of being infected with HIV fueled their discriminatory behaviors toward HIV-positive patients, thereby making the latter group unwilling to publicly seek treatment [26]. Moreover, Khan et al. (2009) noted that *hijra* in Bangladesh do not have access to healthcare facilities due to their stigmatized identities: “If *hijra* suffered from anal STIs, they could not disclose it to doctors. Not only for STIs, because of holding stigmatized *hijra* identity, they had no access to health facilities. They reported hiding their identity while visiting doctors whether in government or private sectors” [28]. Alam (2015) has also observed that stigma and discrimination hinder MSM’s access to public healthcare services in Bangladesh [27]. Moreover, Frances and Aboud (2010) also corroborated the implications of conservative religious and cultural norms with regard to the willingness of community gatekeepers to participate in HIV prevention interventions [29]. These findings underscore the role that several sociocultural and legal factors play in hindering KPs’ access to STI services that are provided by public healthcare facilities.

Although a shift from the DIC-based approach is essential to ensure sustainable STI service delivery, it is equally important to understand the willingness of the KPs to uptake the services that are provided by public healthcare facilities. However, little is known about the willingness of the KPs to uptake these services, thereby resulting in a knowledge gap. In Bangladesh, no formal research study has been conducted till date to explore the willingness of the KP communities to uptake the STI services that are provided by public healthcare facilities [29].

This study aimed to understand and explore the willingness of KPs to uptake the STI services that are provided by public healthcare facilities. Thus, the following research question was proposed: are KP communities willing to uptake the STI services that are provided by public healthcare facilities?

## Theoretical framework

### Social ecological model (SEM)

The nexus between personal and sociostructural factors determines community responses to interventions, community behavior, and service uptake [30–33]. The social ecological model (SEM) is a theoretical framework that is often used to examine personal and sociostructural factors. The SEM has been used to critically analyze health behaviors “in the context of physical, social and policy environments” [34, 35]. It is often used to explain the “complex associations between social (e.g., social networks) and structural (e.g., legalization, access to care) factors, individual practices, the physical environment and health” [35]. It contextualizes “individuals’ behaviors using dimensions such as intrapersonal (e.g., knowledge, attitudes, behavior), interpersonal/network (e.g., social networks, social support), community (e.g., relationships among organizations/institutions), and public policy (e.g., local, state, national laws) to provide a framework for describing the interactions between these levels” [36]. It employs a complex analysis of population health to examine the factors that underlie social inequalities and health disparities. The SEM has been successfully used to examine ecological-level risk factors and vulnerabilities to HIV infection such as structural violence, stigma, discrimination, and willingness to participate in HIV vaccine trials [37–39].

## Materials and methods

### Study design and study sites

To understand and explore the willingness of KPs to uptake STI services that are provided by public healthcare facilities, an exploratory qualitative study was conducted among KPs in three districts in Bangladesh: Dhaka, Chapai Nawabganj, and Munshiganj. Dhaka was selected because it encompasses all three levels of healthcare facilities (i.e., primary, secondary, and tertiary), which coexist with DICs that benefit all KPs. Munshiganj and Chapai Nawabganj district have similar healthcare facilities (i.e., primary and secondary healthcare facilities); however, DIC-based HIV prevention interventions for KPs were absent.

### Study populations: KPs as communities

Classical sociologists and anthropologists define a “community” as a social group that has a collective sense of “we-feeling” and as occupying a specific geographical location. However, this definition has evolved and is indicative of a paradigm shift[40, 41]. In this context, Walkerdine (2012) has postulated that “thinking has fluctuated over time.” This has resulted in a definitional shift from “contained geographical locations” that unify people to “symbolic ties that bind them” even if they are “geographically distant” [42]. Therefore, a community can be defined in terms of systems, and social, virtual, and individual perspectives [43]. Although KPs comprise heterogeneous populations (e.g., PWID, MSM, FSW, *hijra*), scholars have identified them as “communities” for the purposes of research and community mobilization [44–48]. Since KPs are bound by various ties (e.g., shared behavior, symbolic ties, same geographical locations), we conceptualized KPs as “communities” in this study. We have also considered the diversity that exists within the different KP communities (e.g., *hijra* community is different from PWID community or FSW community) to categorically explore and understand the willingness of each of these communities to uptake healthcare services. Data were collected from participants who were 18 years of age or older.

### The operational definition of community willingness

Community willingness is an abstract concept. In this study, it was defined as the willingness of KP communities to be acquiescent and uptake STI services that are provided by public healthcare facilities.

### Data collection and analysis

We conducted 34 in-depth interviews (IDIs) with KPs (Table 1). The participants for the IDIs and focus group discussions (FGDs) were purposively sampled in accordance with the maximum variation sampling procedure in order to facilitate the exploration of the diverse issues that are related to the study objectives (e.g., KPs that receive and do not receive HIV prevention interventions). In addition, we conducted 9 key informant interviews (KIIs) and 11 FGDs with KPs, community leaders, and the leaders of community-based organizations (CBOs). For the KIIs, we used the purposeful sampling strategy that entailed a combination of intensity sampling and critical case sampling. The research participants were selected with the assistance of peer educators (PEs) of DICs and research guides who belonged to the KP communities. PEs are individuals from KP communities who conduct behavior change communication sessions to share STI prevention messages with KP communities, provide them with condoms and lubricants or needle/syringes (for PWID), and refer them to DICs to receive STI services. On the other hand, research guides are community representatives who either visit DICs or are acquainted with PEs and have access to KP communities. We conducted the interviews in locations that were convenient to the research participants (e.g., DICs, homes of the KP community members, spots where the KP communities congregated).

**Table 1:** Socio-demographic status of the 34 KPs participating in the in-depth interviews.

	MSM/MSW			PWID			FSW			Hijra		
	Dhaka	Chapai	Munshiganj**	Dhaka	Chapai	Munshiganj**	Dhaka	Chapai	Munshiganj**	Dhaka	Chapai	Munshiganj**
	(N = 4)	(N = 3)	(N = 3)	(N = 4)	(N = 3)	(N = 0)	(N = 3)	N (3)	(N = 3)	(N = 3)	(N = 3)	(N = 3)
**Age**												
Mean (SD)	24.25 (5.18)	27.00 (5.19)	26.33 (1.52)	41.00 (5.88)	43.00 (12.00)	-	33.67 (4.50)	34.00 (7.90)	25.67 (5.13)	31.33 (5.03)	21.67 (5.50)	29.00 (10.39)
**Monthly Income**												
Mean (SD)	13250 (3947.5)	7666.66 (3785.9)	11666.66 (2886.7)	5500.00 (2516.6)	5333.33 (577.9)	-	22666.66 (7023.7)	9000.00 (1000)	12333.33 (2516.6)	17333.33 (4041.4)	13666.66 (8962.8)	14666.66 (7505.5)
**Education**												
No Education	-	1	-	1	-	-	1	1	-	1	1	-
Primary (1–5)	-	1	-	3	3	-	1	1	2	2	2	3
Secondary (6–10)	3	1	3	-	-	-	1	1	1	-	-	-
Higher (>10)	1	-	-	-	-	-	-	-	-	-	-	-
**Marital Status**												
Unmarried	3	1	3	2	1	-	-	1	1	3	3	3
Married	1	2	-	1	2	-	2	1	2	-	-	-
Divorced/Separated	-	-	-	-	-	-	1	1	-	-	-	-
Widow	-	-	-	1	-	-	-	-	-	-	-	-
**Occupation**												
Sex trade	3	2	1	1	-	-	3	3	3	1	-	-
*Badhai* & sex trade	-	-	-	-	-	-	-	-	-	2	3	3
Small business	-	-	2	-	-	-	-	-	-	-	-	-
Unemployed	-	-	-	1	1	-	-	-	-	-	-	-
Day labor	1	-	-	-	2	-	-	-	-	-	-	-
Begging	-	-	-	2	-	-	-	-	-	-	-	-
Agriculture	-	1	-	-	-	-	-	-	-	-	-	-
**Intervention**												
HIV intervention coverage area	4	3	-	4	2	-	3	2	-	3	3	-
Within intervention	4	3	-	4	2	-	3	2	-	3	3	-
Out of intervention	-	-	3	-	1	-	-	1	3	-	-	3

** There is no intervention for any KPs in Munshiganj and research team was unable to find any PWID.

Data were collected using semi-structured guidelines that have been developed based on the findings of studies that were conducted among these communities and existing community willingness models [29, 49, 50]. The semi-structured guidelines included questions such as the following: “Did you visit a public healthcare facility for the treatment of STI? What was the reason for visiting the public healthcare facility? What was your experience there? What challenges did you face while visiting those facilities? Why do you think you had faced those challenges? Was there any positive experience in those public health facilities? What were those? Based on your experience, how your positive experiences can be utilized to receive STI services from public healthcare facilities? What is your opinion about the feasibility of STI services provided from public healthcare facilities for KPs?”

The sample inclusion criteria were as follows: (1) KP community members (e.g., MSM, *hijra*, PWID, FSW) who are18 years of age or older and (2) those who provided informed written or verbal consent for participation in the study. Participants who were unwilling to provide written consent provided verbal informed consent, which was recorded using a digital recorder. The study did not include any minors (i.e., < 18 years of age). Participation in this study was entirely on a voluntary basis, and a consent form that was approved by the Research Review Committee and Ethical Review Committee of International Centre for Diarrhoeal Disease Research, Bangladesh (icddr,b) was read to the research participants before conducting the interviews or FGDs.

Digital recorders were used to record the interviews and FGDs. However, in some cases, the informants were uncomfortable about being recorded; in such cases, we made handwritten notes. The hand notes were elucidated at the earliest possible time, particularly before conducting the next interview. During FGD sessions, in addition to digital recordings of the proceedings, one of the team members was assigned to prepare handwritten notes. After each IDI, KII, and FGD, the transcripts were prepared by listening to the recordings line-by-line and incorporating field notes when necessary.

The trained members of the research team (including the research guides) conducted repeated observations at the public healthcare facilities. They sought to observe events such as the presence of KPs at the public healthcare facilities, behaviors of the hospital staff toward KPs, interactions between KPs and those who are involved in service delivery, and the reaction of the other patients in the presence of KPs. In order to observe these phenomena carefully and systematically, we developed and utilized semi-structured guidelines for observations. Similar semi-structured guidelines were used for interviews and FGDs. The semi-structured guidelines ensured that the researchers observed events consistently without limiting the events that were under observation. The team observed events with an open mind to accommodate issues that were perceived to be pertinent to the research objectives. The observation was an ongoing process, and it was conducted alongside other qualitative data collection methods. The research team members who were involved in observation prepared detailed write-ups based on their detailed observational notes on a regular basis and shared these notes with the other team members. The observational notes included objective-specific descriptions and subjective interpretations of their observations.

We undertook data collection and analysis concurrently because they are not only an ongoing and reflexive processes but they also facilitate the identification of data saturation [51, 52]. The data were thematically and contextually analyzed using a line-by-line manual data analysis procedure. The data were coded into themes and subthemes. We developed a structural coding method by designing a joint or collaborative coding framework in lieu of the solo coding framework [53]. During primary analysis, we identified emerging themes and subthemes, and further analyzed their contexts and meanings. We also identified gaps in the data and issues that had not been previously covered. Atypical data were not overlooked. Instead, they were further explored, analyzed, and presented as findings. As has been suggested by many qualitative researchers, the researchers of the present study the maintained field diaries to document detailed field-level observations and interpretative findings, which were analyzed in a manner that was similar to what was followed for interview transcripts [54–57]. The research team interpreted and exchanged findings during peer-debriefing meetings. This nurtured inter-subjective interpretations and facilitated the modification of data collection tools [51]. A range of strategies was utilized to ensure scientific rigor (i.e., validity and reliability), primarily by means of triangulation (i.e., relying on more than one data source, method, investigator, and analytic approach)[58].

We used the “Consolidated criteria for reporting qualitative studies (COREQ): 32-item checklist” to report the findings (S1 Table) [59]. In addition, we did not attempt to generalize the findings to a wider population. Instead, we obtained a systematic and in-depth understanding of the research question. Since the existence of multiple realities lie at the core of qualitative research, we investigated these realities by searching for disconfirming evidences. We only incorporated findings when they “survived serious attempts to falsify it” [58, 60]. We have strengthened the validity and reliability of the findings and minimized internal bias by providing “thick descriptions” (i.e., explicit and detailed descriptions) of the research process and findings [58, 61].

This study was approved by the Research Review and Ethical Review Committees of icddr,b.

## Results

**Fig 1:**
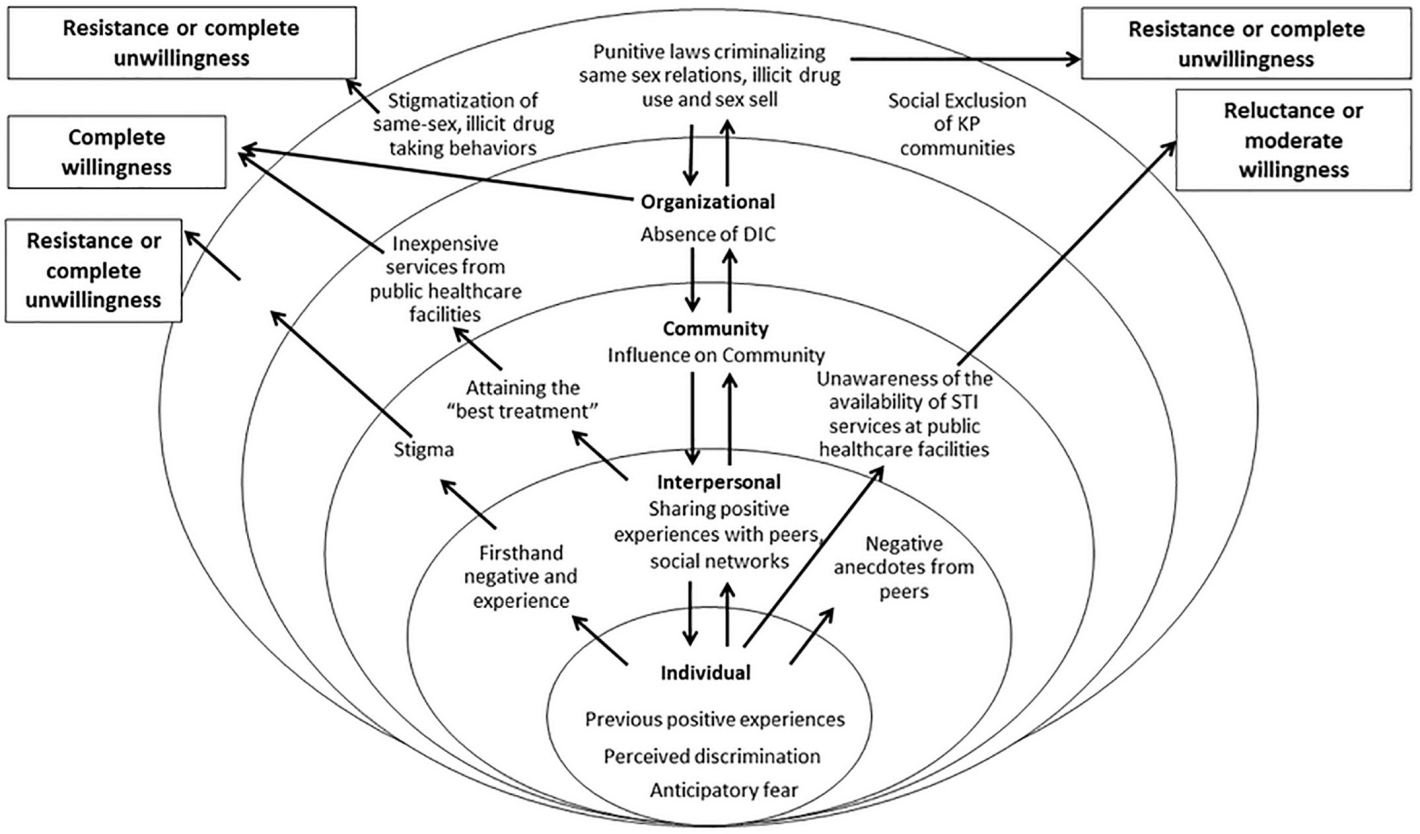
SEM framework on community willingness.

Based on the thematic and contextual analysis of the findings, three types of “willingness” emerged during ongoing data analysis: (1) complete willingness, (2) reluctance or moderate willingness, and (3) resistance or complete unwillingness. These three types of willingness are illustrated in the diagram that is presented in Fig 1.

### (1) Complete willingness

Complete willingness refers to the amenability of KPs to uptake STI services that are provided by public healthcare facilities. This sentiment was influenced by positive experiences and perceptions at the individual, interpersonal, community, organizational, and structural levels, provided that they either had no other viable healthcare-seeking option or had previous positive experiences. Their beliefs that they could attain the “best treatment” from public healthcare facilities optimized their motivation to visit these facilities.

Barring street-based female sex workers (SFSW) and PWID, most FSW, MSM, and irregular PWID (i.e., PWID who occasionally inject drugs), who are typically not identifiable based on their outward appearances, were motivated and willing to seek treatment for STIs from public healthcare facilities. The research participants conveyed this willingness either because they had no other feasible option, or they believed that they could attain the best treatment at a minimal cost from public healthcare facilities. Very few of them had previous positive experiences when they had sought STI services from public healthcare facilities. One of the FSW participants made the following remark:

Some of us usually visit government hospitals since doctors of those hospitals are renowned and better than the doctors [Medical Assistants] of the DICs. If someone, say *madam* or *dalal* [broker of sex trade], accompanies us, and we behave well with them [doctors], the doctors of the government hospitals behave well with us, even after knowing that we are sex workers.

(FSW, 32 years old, FGD, Dhaka, Intervention for KPs)

I visited *Sadar* hospital [public hospital in Munshiganj district] once when I was suffering from a sexual problem [STI] … I had a good relationship with a female support staff (*aya*) over there. I mainly prefer visiting there because I can receive treatment at minimal cost, and I get free medicines.

(SFSW, 30 years old, IDI, Munshiganj)

Similarly, MSM and male sex workers (MSW) in Munshiganj district, albeit very few, reported that they were more inclined to visit public healthcare facilities, especially because it is a more inexpensive option when compared to visiting a private hospital or DICs in another district. Furthermore, it is more likely that their services will be expedited if they have “good connections with the support staff of *Sadar* [district] hospital.” Similarly, one of the very few *hijra* who expressed complete willingness recounted a situation in which her *guru* (i.e., leader of *hijra* community) was able to gain the necessary medical support due to her “connections” with a support staff member. She attributed their positive experiences to the fact that the KPs in Munshiganj and all other districts were accompanied by or received support from someone who worked within the hospital when they visited public healthcare facilities. Similarly, a Dhaka-based *hijra* recounted that not only was her physician amenable to treating her STI, but he also examined her anus in a private environment and behaved cordially with her. She described her experience in the following manner:

The doctor behaved well and prescribed me medicines after the physical examination. There was a provision for screening my anus at a place covered with white curtains (*sadaporda die ghera*). The doctor examined my anus in the examination corner of the room and prescribed the treatments. He was different from the other doctors I have met before… he listened and paid attention to my problems. I might visit this hospital again if there are doctors like him.

(*Hijra*, 30 years old, IDI, Dhaka)

While most participants of the PWID community recalled negative experiences with regard to their visits to public healthcare facilities, there were very few noteworthy positive reviews. For instance, very few PWID in Chapai Nawabganj district recounted that they were able to freely express their problems to the physicians. One of them made the following observations: “I faced no difficulty in expressing my problem. While expressing this problem, I brought my mouth near the doctor’s ear. He was quite okay with this and listened to me" (PWID, FGD, Chapai Nawabganj). This PWID alluded that physical touch and proximity symbolize an emotionally intimate interaction between the doctor and patient.

### (2) Reluctant or somewhat willing

Reluctant or somewhat willing was defined as the state of not being willing to uptake STI services from public healthcare facilities due to the individual, interpersonal, community-level factors including perceived negative anecdotes from peers, perceived discrimination and anticipatory fear by KP communities; and community-level barriers such as unawareness of the availability of STI services at public healthcare facilities.

#### Perceived discrimination and anticipatory fear

KPs that perceived prejudices and discrimination against them reported that their internalized fear of facing discrimination was another barrier to visiting public healthcare facilities. Even though the KPs received STI services, they reported that they were not treated “properly” and in a “sensitive manner” by the physicians, nurses, and other staff, who are collectively known as healthcare providers (HCP). These sentiments are the possible outcomes of perceived discrimination. Several KPs reported instances in which they felt that their physicians diagnosed them as quickly as possible so they could leave the hospital premises immediately and minimize the time that they had to spend with them. As a result, a few KPs felt like they were not welcome to seek STI services at the healthcare facilities.

A positive doctor-patient interaction requires the doctor to attentively listen to the patient’s problems. This can be quite difficult to practice in public healthcare settings due to a limited number of physicians who provide services to a large number of patients. Nonetheless, a few KPs reported that they felt that the physicians not only failed to listen to them but also did not value them.

In addition, the anticipatory fear of discrimination and antagonizing behaviors of HCPs due to stigma, social exclusion, and existing punitive laws that criminalize same-sex behaviors, effeminate MSM (i.e., *kothi*) and MSW reported that they felt “ashamed” about their same-sex sexual relationships. As a result, they often refrained from disclosing their sexual orientation. Hence, instead of admitting that they have anal STIs and engage in sex with other men, they fabricate other conditions such as hemorrhoids or anal fissures that result from constipation. In this regard, one MSW made the following statement:

I visited the doctor since the skin of the anal canal tore (*baatli fete giache*) while having sex with a man. Then, the doctor asked, “How did you get this problem?” Since I know that the doctor will not blame me for anal fissure, I told him, in a hushed tone, that I have an anal fissure. I replied that I have constipation, and this problem occurred while defecating. I felt ashamed and feared that the doctor may scold me upon hearing my sexual practices. I decided to hide the real reason.

(MSW, 40 years old, FGD, Munshiganj)

This particular participant believed that lying about his condition would allow him to have a “peaceful” interaction with his physician. However, KPs should ideally be able to openly express their problems without the anticipation of negative behaviors or the fear that they will be perceived differently because of their unconventional and nonconforming practices. Thus, such anticipatory fears served as one of the major barriers that interfered with the willingness to seek medical services from public healthcare facilities among hidden and marginalized groups of KPs such as MSWs and *hijra*.

Most of the male and female sex workers chose to not reveal their profession to their physicians because commercial sex work is stigmatized. Therefore, they believed that their physicians may pass judgmental or hurtful comments. When they did disclose their professions, sex workers believed that the doctors were silently judging them and avoiding them. One of the SFSWs from Chapai Nawabonj explained her experience at the district hospital in the following manner:

At first, I first said that I had white discharge and itching on my genital area. The doctor behaved well and listened to me attentively. I thought I had been suffering from a complicated disease due to my profession. Therefore, I disclosed my profession to get proper treatment. However, the doctor stopped asking questions and quickly prescribed me after knowing that [about my profession]. Although he did not misbehave, and he still provided treatment, I think he stopped talking to me just because I disclosed my identity… it felt like the doctor was avoiding me.

(SFSW, 32 years old, FGD, Chapai Nawabganj)

In this case, although the physician treated the SFSW and neither misbehaved nor expressed any judgmental attitude toward her, she felt that the doctor was avoiding her and treating her differently merely because she divulged her profession to him.

Further, PWID reported that they faced serious repercussions of perceived discrimination, including low self-esteem. In a KII, one of the CBO leaders offered the following explanation:

Due to taking injectable drugs for a long time, most PWID lose the ability to value their life, and confidence deteriorates completely. Besides, not bathing for a long time, not brushing or cutting hair, and dirty attire collectively produce odor, which creates a divide with general people. General people do not want to wait in the queue with them and try to distance themselves from PWID, which adds to their low self-esteem. Thus, PWID do not feel comfortable standing alongside general people in the government hospital, and they feel like they would be scolded.

(CBO leader-PWID, KII, Dhaka)

The PWID community was entrapped in a vicious cycle, whereby the members of their society shunned them because of their poor hygiene and shabby attire. In turn, they felt as though they were rejected by society, and they consequently lost the ability to value themselves. Because they believed that other patients may feel disgusted by them, they felt antsy about occupying the queue alongside these patients. Taken together, these factors caused them to experience a sense of perceived discrimination, which in turn made PWID believe that it is not worth the extra effort to seek healthcare, especially because it was very unlikely that someone would be able to adequately address their needs.

This specific finding is a clear indication that internalized negative feelings, perceived stigma, and anticipatory fear can have an impact on the willingness of PWID, FSW, MSWs, and *hijra* to seek STI services from a public healthcare facility.

#### Lack of awareness about the availability of STI services at public healthcare facilities

Across all the districts, KPs, especially MSWs, *hijra*, and SFSWs, were unaware of the availability of STI services at public healthcare facilities. This was true even in Munshiganj where there were no available HIV prevention interventions. However, they were aware of the STI services that were provided through the DICs in other districts, which they had heard about from their peers. Accordingly, one of the study participants made the following observation:

Often, I visit Dhaka for many other purposes. I know in Dhaka Medical [Dhaka Medical College and Hospital] there is a department where doctors provide services only for the sexual disease [STIs]. There are DICs for us (*hijra*) in Dhaka and Narayanganj districts as well. To my knowledge, there is no such service in our Munshiganj district hospital. I don’t know whether such services are available here or not. Therefore, I did not think of going there to treat my sexual problem.

(*Hijra*, 30 years old, IDI, Munshiganj)

I am aware that services like delivery [vaginal or delivery by operation of child] and treatment of these issues are available at the government hospitals. On the other hand, I don’t know whether the treatment for STI is available there… I’ve never heard of this.

(SFSW, 26 years old, IDI, Dhaka)

### (3) Resistance or complete unwillingness

Resistance or complete unwillingness refers to a state in which one strongly opposes and expresses an unwillingness to uptake the STI services that are provided by public healthcare facilities due to firsthand deleterious experiences that are considered to be negative and undesirable from the participants’ perspectives. These KPs expressed various reasons for their resistance to visit public healthcare facilities that were rooted at the individual, interpersonal, community, organizational, and structural levels. Structural barriers included the criminalization and stigmatization of same-sex sexual and illicit drug-taking behaviors. Most of the KPs were resistant or completely unwilling to uptake STI services that were provided by public healthcare facilities.

#### Previous negative experiences with HCPs and other human resources for health (HRH) at public healthcare facilities

Most of the HCPs and other HRH (e.g., physicians/doctors, nurses, technologists, storekeepers, pharmacists, ward boys, guards, cleaners, members of lower subordinate staff [MLSS]) dubbed the behavior of KPs as *onoitik achoron* (meaning “immoral behavior”) and *ossavabik* (meaning “abnormal” behavior). These views were engendered by societal constructs and collective norms and values that are rooted in the sociocultural and religious contexts of Bangladesh. These include the stigmatization of illicit drug use and commercial sex work as well as the criminalization of same-sex relationships. In turn, these sentiments manifested themselves in the manner in which HCPs and HRH treated KPs. This made them resistant to visit public healthcare facilities. For example, one of the MSWs made the following observation:

I went to "X” hospital to treat my *batli bila* (anal rupture). Doctor asked me the reason behind my problem. I had no choice but to disclose that I had anal sex with a male. After knowing my sexual relationship with a male partner, he asked me, “Being a male, why do you have sex with a male? This is an extremely bad practice; you should stop such sinful acts and relationships.” I went there to receive treatment, not to get advice on my sexual preferences. I felt ashamed, and I might not visit there again.

(MSW, 38 years old, IDI, Dhaka)

Similarly, a female PWID (who was also an FSW) reported that the physician posed a series of intrusive questions about her lifestyle. She felt that the physician was more invested in offering unsolicited advice rather than alleviating her problems. Accordingly, the FSW narrated her experience in the following manner:

The doctor said, “Why do you do such work (selling sex)? Why do you inject drug? Does any woman do it? You people are taking drugs! Are you good people? … Give up all of it.” Why did the doctor give me these suggestions? I know my life very well. I went there to receive treatment, not to take such advice from the doctor who cannot solve my problems. Giving advice is easy, but can anyone solve my problems? I felt angry and ashamed, and I won’t go there again to just to get embarrassed even more.

(FPWID/FSW, 31years old, IDI, Dhaka)

#### Disrespectful and judgmental behavior by HCPs and other HRH at public healthcare facilities

Most KPs who visited public healthcare facilities reported that they had faced deleterious, disrespectful, judgmental, and quite often antagonistic behaviors from HCPs and HRH. Their unpleasant experiences entailed not only verbal disputes but also neglectful and antagonistic behaviors that diminished KPs’ morale and made them resistant to uptake STI services that were offered by public healthcare facilities. The KPs faced various forms of disrespectful and judgmental behaviors that were shaped by not only individual and community-level barriers but also structural barriers. More specifically, in addition to HCPs’ refusal to provide treatment and antagonistic attitudes toward the KPs, they also displayed behaviors that were molded by structural factors such as punitive laws against illicit drug use and same-sex relationships. Hence, when KPs visited healthcare facilities to seek STI services, they had to endure the threat of being reported by HCPs to law enforcement agencies for their identities and behaviors. This made them completely unwilling to revisit public healthcare facilities because they feared that they might have to face severe legal ramifications.

One of the participants who belonged to the PWID community complained that he had to dress and drain his own abscess because no one at the facility wanted to help him, despite his persistent pleas. He recounted his experience in the following manner:

They (nurses/ward boys) did not want to come near me because my abscess smelled bad… I smelled bad. I requested several staff; however, no one came to help me. I was asked to manage abscess by myself on the verandah. They pointed fingers at me, used bad terms to indicate me, and avoided me, as if I were not human. It was too much! I would rather die than ever visit there again.

(PWID, 27 years old, IDI, Dhaka)

Likewise, another PWID in Dhaka, who had a similar negative experience of being humiliated and neglected by HCPs, was so frustrated that he exclaimed, “Even if my deceased father asked me to go there [public healthcare facility], I will still never visit there!” Moreover, HCPs labeled KPs with derogatory terms and shunned them because of their identities and the practices that characterized them as KPs. In some cases, they even refused treatment to PWID because they inject drugs. For example, a PWID from Chapai Nawabganj who sought medical assistance for an ulcer recounted the following experience:

I found two nurses. First, I showed them the ulcer on my leg and requested treatment. They immediately told me to leave the room. They said they would not offer treatment to *heroinchies* (a derogatory term used to address illicit drug users) like me. I had to come back without treatment.

(PWID, 39 years old, IDI, Chapai Nawabganj)

Most MSM and MSWs reported that they felt apprehensive about visiting public healthcare facilities. Further, partaking in same-sex relationships fueled their fear of legal repercussions. They also reported that they had received *threats* rather than *treatments* from their physicians. One of the MSM recounted the following experience of being threatened because of his same-sex sexual behaviors:

Once I had to visit district hospital to treat anal discharge. The doctor wanted to know about my sexual practices after hearing the anal problem. I had to disclose my sexual practices. He said that male-to-male sex is a crime. Then, he threatened to hand me over to the police. I waited no longer in the doctor’s room. Instead, I hurried out of his room without taking the prescription. Do you think I will visit him again after this incident? I shared this experience with my *gotiya* [a term that means “sister”; it is used by the *kothi* and *hijra* to refer to other *kothi* and *hijra*], and they are scared too.

(MSW, 37 years old, FGD, Chapai Nawabganj)

In addition to these fears, *hijra* experienced another layer of complexity. Many *hijra* reported that they were not able to find a queue for their gender. They were also mocked by other patients and HCPs, and had been given the “cold shoulder.” Overall, HCPs and other HRH lacked an understanding of the gender identities and sexualities of *hijra* community that was reflected in their behaviors. In this regard, one of *hijra* offered the following explanation:

I went to “X” hospital since I had itching outside my genital area. I bought a 10-taka [equals to 0.012 USD] ticket and stood in a queue. There are designated queues for women and men. I headed to the women’s line. The women moved away and stood elsewhere, and men did the same. And the doctor? The doctor watched me from afar and anxiously asked, “Who is this person? What am I going to do with him?” As a human being, I have a sense of decency too! You get scared of me just by looking at me? Have I even done anything to make you feel scared? I am not someone you should be scared of. Every day, men and women visit this hospital and leave with the treatment they need, but there’s no option for *hijra* here. Neither am I able to enter nor am I able to consult with the doctor. They don’t bother to treat us the same way they treat, listen to, and advise other patients. At this point, I feel ashamed because what’s the point in discussing my illness if no one can tolerate me?

(*Hijra*, FGD, Dhaka)

Most of the FSWs who visited the public healthcare facilities reported that they had faced antagonistic behaviors from HCPs and the other HRH. A few FSWs reported that they were being abused in different ways. This made them resistant to the idea of revisiting the public healthcare facilities. One of the SFSWs described her experience in the following manner:

I was facing some problems [STI]. I went to “X” hospital. I work [sell sex] at the street, and my attire was shabby. While entering the hospital, I overheard someone saying, “Look! After doing dirty work (*akamkukam)* all night, she got the disease. And now, she is here to visit the doctor!” When I told the (male) doctor about my problem, he asked me, “How did you get this disease?” So, I told him about my profession: that I work as a sex worker. Suddenly, his reaction changed. He told me, “Shame on you! You are a bad girl, and what you do is immoral. Of course, you will be getting this disease. Who else would? You don’t deserve to get treatment.” Later, he told me to lie down on a bed and told me that he needs to examine the infected place. After I laid down, he started touching my breasts and other intimate body parts. I begged him to stop. He wrote something on the prescription and told me to take medication. Look, I choose this profession (sex work) merely for survival. I went to the hospital to get treatment, not for other purposes or for being abused.

(SFSW, 28 years old, IDI, Dhaka)

## Lack of privacy

To receive necessary treatment, KPs must disclose their histories. However, to circumvent situations in which fellow patients may scrutinize or mock their behaviors, they prefer to access a more secluded environment where their privacy is more likely to be maintained. Several KPs unfavorably compared the degree of privacy in public healthcare facilities to their respective DICs. They opined that they preferred the latter because they could easily disclose their details to the HCP within a closed-door setting. On the other hand, in public healthcare facilities, the doors were observed to be constantly open, and the patient queues overflowed to such an extent that other patients could hear the conversations that were exchanged between the physician and patient. One of *hijra* who participated in this study described her experience in the following manner:

For me, it is not possible to express my sexual problems in front of several people. Since the door is open, other patients can hear my private information. Often, people I know stand there. On the other hand, we do not face such problem in DIC as the doctor (referring to the Medical Assistant) provides services in a closed-door room, where we can express everything. I cannot imagine such services in the government hospital.

(*Hijra*, 32 years old, IDI, Chapai Nawabganj)

Since multiple physicians occupy a room and concurrently interact with other patients, it is difficult for patients to freely divulge personal information. KPs fear that other patients may mock them or spread rumors about them to other people in the area. Therefore, they lie about their condition to physicians. When compared to the other KP communities, this sentiment is more amplified among MSWs because of their perceived fear of being reported to law enforcement agencies. In this regard, one of the MSWs from Chapai Nawabganj made the following observation:

When I entered the doctor’s room, I found several people besides the doctor, such as medical representatives, intern medical assistants, etc. Often, the patient queue overflows to the doctor’s room as the door is always open. Disclosing private matters in front of several people is difficult. I finally went in to get my anal problem treated. But I could not express my problem clearly because I was feeling embarrassed and due to shyness. Therefore, I said that I had back pain. At DIC, it is not a problem to share anything as the door of the doctor’s room always remains closed while treating patients.

(MSW, 35 years old, IDI, Chapai Nawabganj)

## Discussion

Most KPs were either “reluctant” or “resistant” to seek STI services that were provided by public healthcare facilities. None of the KPs were avidly willing to uptake STI services that were provided by public healthcare facilities. In all the three districts, some MSM, FSWs, and *hijra* felt uncomfortable about disclosing their sexual practices and professions to their physicians due to fear of identity disclosure, anticipated stigma, and adverse legal consequences. Similarly, previous studies that have been conducted by Khan et al. (2009) and Chakrapani et al. (2011) have examined *kothi*’s qualms about being reported to the authorities as well as demeaning behaviors such as a lack of privacy and refusal of treatment that some *kothi* have been subjected to [28, 62]. Khan et al. (2009) also reported that *hijra* were hesitant to disclose their health issues and encountered “abusive behaviors” in healthcare settings [28]. A few other studies have also reported that MSM and transgender women face similar struggles when they avail STI and HIV-testing services, which render them resistant to the uptake of HIV-testing services [63–66]. MSM and MSWs, particularly *kothi*, fear being reported to the legal authorities due to the criminalization of same-sex sexual behaviors in Bangladesh. However, our study findings reveal that *hijra* also face this problem not only due to their sexual behaviors but also due to the lack of understanding about their gender identities on the part of HCPs and other HRH.

Similarly, FSWs reported that they were abused in many ways and that they were given unsolicited advice and shamed for their professional choices and practices. Similarly, Wahed et al.’s (2017) study also noted that unfriendly behavior on the part of HCPs is a barrier that precludes FSWs from availing sexual health services [67]. Similarly, a Nepal-based study also identified a lack of privacy, judgmental attitudes of service providers, and discrimination as deterrents that impede the utilization of sexual health services among FSWs [68]. However, our study also evinced that FSWs faced sexual harassment in public healthcare facilities; this finding is a novel contribution to the existing literature.

A few SFSW were willing and are at complete willingness state to visit healthcare facilities. The findings of our study can be contrasted against the findings of a few other studies that have been conducted in other countries, where all categories of FSWs reported that their primary concerns about visiting public healthcare facilities were attributable to the lack of privacy and fear of being identified as sex workers [10, 62, 63]. Nonetheless, this finding is similar to Logie et al.’s (2010) observations that highlighted structural issues such as privacy being a major concern among MSM and transgender women in Jamaica [65]. However, our study findings suggest that the fear of being identified as a sex worker is more prominent among SFSWs than other FSWs. Indeed, due to their “unusual attire and unpolished appearance and mannerisms,” it is easier for HCPs and other HRH to identify them as sex workers. On the other hand, residence-based female sex workers (RFSW) in Bangladesh were found to typically embody a more “refined outward appearance.” Therefore, they may be able to easily blend in with other female patients. Nevertheless, FSWs who belong to both these categories were anxious about attending public healthcare facilities. They also dreaded the possibility that someone else might find out about their profession (i.e., sex workers) and consequently treat them in an unpleasant manner.

PWID generally had low self-esteem because they were denied STI services at public healthcare facilities, called derogatory names, and/or antagonized for their shabby physical appearance. This propagated a vicious cycle that resulted in a refusal to seek treatment. Their low self-esteem, perceived discrimination, and the fear of being antagonized by HCPs have also been documented in previous studies that were conducted in developing and developed countries [69–72]. Specifically, perceived discrimination hinders KPs’ unwillingness to seek STI services from public healthcare facilities. A few other previous studies have also indicated a potential link between perceived discrimination and poor health outcomes [73–77].

In this article, applying the SEM framework, we attempted to explore the willingness of the KPs to visit public healthcare facilities and the underlying reasons for different categories of willingness. Within the framework of SEM, our findings highlight the unwillingness of the individual KPs to seek STI treatment from public healthcare facilities, and their difficult interactions with a stigmatizing, discriminatory and incompetent healthcare system. Structural factors, such as the all-encompassing stigma and discrimination the KPs face in healthcare settings (e.g., primary, secondary and tertiary levels) also hamper their willingness to visit the public healthcare facilities. Punitive laws against same-sex relationships and sex work, as well a lack of institutional guidelines on which queue *hijra* can choose to stand in the outpatient department contribute to KP’s decision on not wanting to come back again.

In essence, our observations resonate with past findings in that KPs in Bangladesh exhibit a general lack of willingness to visit public healthcare facilities in order to avail STI services. However, none of the studies that have been conducted in LMICs have reported that KP communities undergo pleasant and hassle-free experiences when they visit hospitals. Nevertheless, our findings suggest that not all of their experiences at public healthcare facilities negatively impact their willingness to uptake STI services. Hence, efforts to enhance the willingness of KPs to avail STI services at public healthcare facilities must aim to both enhance the positive aspects and mitigate the negative aspects of their encounters with HCPs and other HRH.

## Conclusion

**Fig 2:**
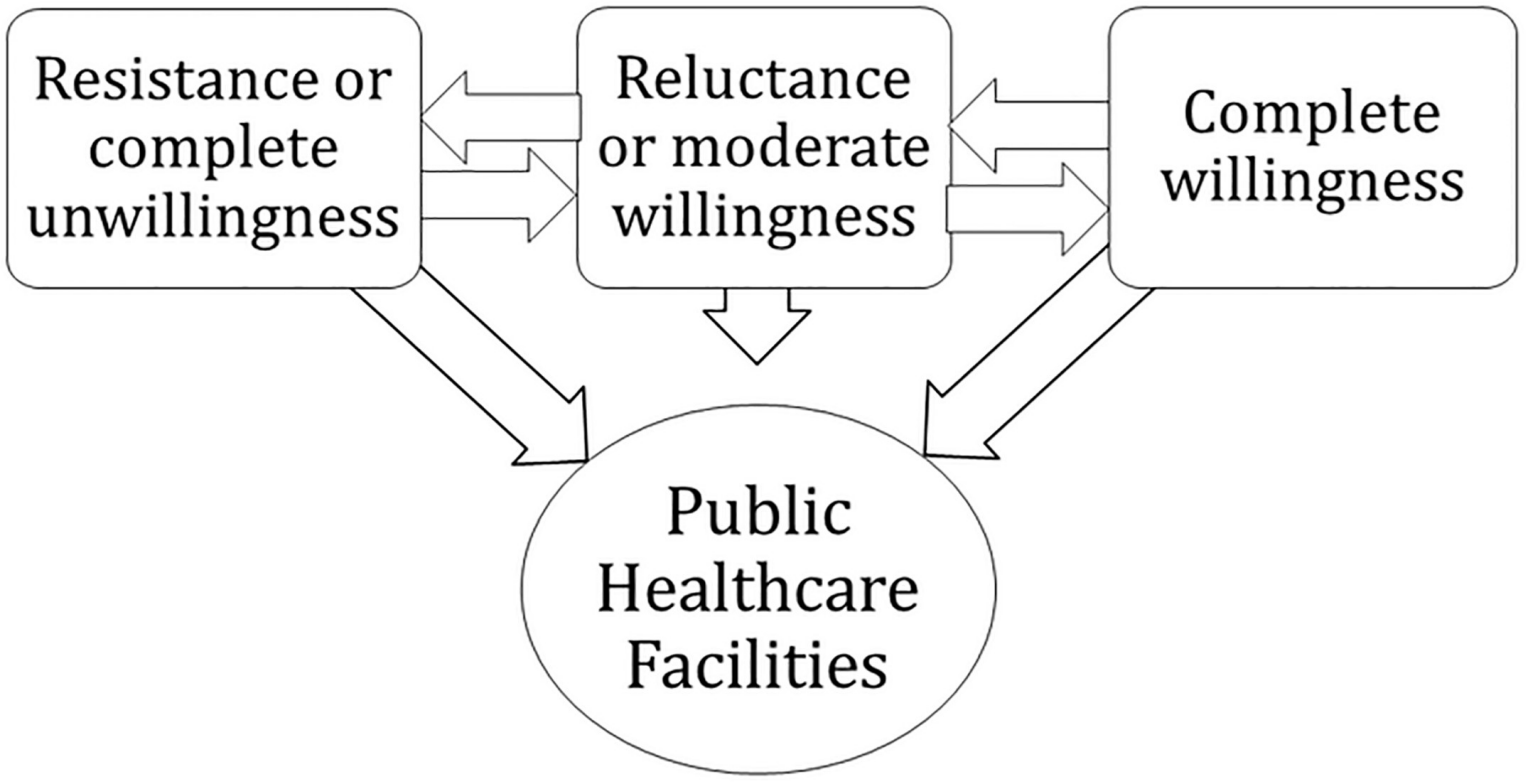
Continuum of community willingness.

In this article, we used the SEM theoretical framework, which entails individual behaviors as well as structural factors (e.g., experiences of KPs when they visit public healthcare facilities, hidden nature of KPs, stigma) to contend that KPs’ willingness to uptake STI services that are provided by public healthcare facilities does not depend solely on individual or community experiences. Instead, it is linked to a nexus between sociostructural factors and health inequalities. KPs’ willingness to uptake STI services predominantly can be conceptualized as a continuum that ranges from “resistant” to “completely willing” (Fig 2). Resistance toward the uptake of STI services that are provided by public healthcare facilities was mainly exemplified by a substantial proportion of the participants who were completely unwilling to visit public healthcare facilities due to their firsthand adverse experiences with the unsympathetic and often antagonistic healthcare providers. On the other hand, very few participants appeared to be situated at the “completely unwilling” end of the spectrum based on their positive firsthand experiences and anecdotal reports from peers. However, since this is a continuum, some participants belonged to neither extreme. Nevertheless, they were considered to be “resistant” because they were hesitant to uptake services unless certain conditions were fulfilled.

The entire spectrum of community willingness primarily relies on the preparedness of the public healthcare system to administer STI treatments. It is also important to note that this spectrum is dynamic. This indicates that KPs can vacillate between feeling “resistant” and “completely unwilling” depending on their own and peers’ experiences. For instance, it is possible for “reluctant” participants to gradually start feeling “completely unwilling” or “resistant” depending on the nature of the experiences that they are subjected to when they revisit public healthcare facilities.

All the KPs expressed a willingness to revisit public healthcare facilities if (a) the HCPs and other HRH are sensitized about the culture and practices of KP communities, (b) efforts are made to protect their privacy, and (c) changes in discriminatory behavior and stigmatizing attitudes toward KPs are ensured. In order to achieve this, training programs must be conducted at public healthcare facilities, and the health system as a whole must be strengthened. Policy and practice-related changes that aim to enhance confidentiality and reduce discrimination in public healthcare facilities are also vital. The findings also underscore the need for interventions that address sexual identity-related stigma in public healthcare settings in order to augment KPs’ willingness to visit these facilities. The findings also revealed that KPs are unlikely to seek STI services that are provided by public healthcare facilities if structural factors (e.g., stigmatizing, discriminatory, and the incompetent healthcare system; punitive laws against same-sex sexual behaviors and sex work) and all-encompassing stigma remain unaddressed.

Additionally, community mobilization must be promoted by empowering and involving influential members of the KP community (e.g., CBO leaders, *hijra guru*). As posited by Thurman et al. (2000), “Communities are always changing, adapting [and] growing. They are ready for different things at different times” [44]. Hence, depending on the changes that occur in the KP community, the public healthcare system must be resilient and prepared to evolve in order to address the emerging needs of KPs by adopting a public-private partnership approach.

## Supporting information

S1 Table The willingness to receive sexually transmitted infection services from public healthcare facilities among key populations at risk for human immunodeficiency virus infection in Bangladesh: A qualitative study. (DOCX)
