# Supplementary material for: The willingness to receive sexually transmitted infection services from public healthcare facilities among key populations at risk for human immunodeficiency virus infection in Bangladesh: A qualitative study
Source: PLoS One. 2019 Sep 4;14(9):e0221637. doi: 10.1371/journal.pone.0221637 (PMC6726367; doi:10.1371/journal.pone.0221637)
Supplement: S1 Table — (DOCX) [file pone.0221637.s001.docx]

**S1 Table. COREQ checklist (1)**

For:

**The willingness to receive sexually transmitted infection services from public healthcare facilities among key populations at risk for human immunodeficiency virus infection in Bangladesh: A qualitative study**

| **Topic** | **Item No.** | **Guide Questions/Description** | **Details/ reported on page no.** |
| --- | --- | --- | --- |
| **Domain 1: Research team and reﬂexivity** | | | |
| *Personal characteristics* | | | |
| Interviewer/facilitator | 1 | Which author/s conducted the interview or focus group? | Gorkey Gourab  Mohammad Niaz Morshed Khan  AM Rumayan Hasan  Golam Sarwar  Page no. 1 |
| Credentials | 2 | What were the researcher’s credentials? E.g. PhD, MD | Gorkey Gourab (GG), MSS, MPH  Mohammad Niaz Morshed Khan (MNMK), MSS, MPhil, MPH, PGD  AM Rumayan Hasan (AMRH), MSS, MPH  Golam Sarwar (GS), MBBS, MPH, PGD  Samira Dishti Irfan (SDI), MPH  Md. Masud Reza (MMR), MSc  Tarit Kumar Saha (TKS), MBBS  Lima Rahman (LR), Master in Population and Reproductive Health Research, MBBS  AKM Masud Rana (AKMMR), PhD  Sharful Islam Khan (SIK), MBBS, MHSS, PhD  Non-author data collectors held Master’s degree and above. |
| Occupation | 3 | What was their occupation at the time of the study? | GG, MNMK, AMRH, GS, SDI, MMR, AKMMR and SIK were full time researchers with many years of experience in the field of HIV and AIDS. All of them worked at International Centre for Diarrhoeal Disease Research, Bangladesh (icddr,b) and were also involved in implementing intervention on HIV for the key populations (KP) at risk of HIV.  TKS worked for the National AIDS/STD Programme for the Government of Bangladesh. TKS now works for Institute of Public Health (IPH), Directorate General of Health Services (DGHS), Ministry of Health and Family Welfare, Government of the People's Republic of Bangladesh.  LR worked on HIV/AIDS Program at Save the Children, USA. TKS guided the National response to HIV and AIDS in Bangladesh, and LR worked on implementing intervention on HIV for KP. Both of them had many years of research and program implementation experience in the field of HIV and AIDS.  Non-author data collectors were full-time researchers.  Page no. 1-2 |
| Gender | 4 | Was the researcher male or female? | Gorkey Gourab (GG), male  Mohammad Niaz Morshed Khan (MNMK), male  AM Rumayan Hasan (AMRH), male  Golam Sarwar (GS), male  Samira Dishti Irfan (SDI), female  Md. Masud Reza (MMR), male  Tarit Kumar Saha (TKS), male  Lima Rahman (LR), female  AKM Masud Rana (AKMMR), male  Sharful Islam Khan (SIK), male  Among non-author research guides, two were female, two were male, and one was transgender women (known as *hijra*). |
| Experience and training | 5 | What experience or training did the researcher have? | GG, MNMK, SIK: Extensive qualitative research experience on HIV and AIDS among key populations (KPs). In addition, GG received training on qualitative research methods and analysis from Vanderbilt University, USA, and the University of Melbourne, Australia.  MMR, AKMMR, TKS, LR: Experience on quantitative and mixed method research, key populations, HIV and AIDS.  AMRH: Experience on qualitative research, and HIV and AIDS.  GS and SDI: Qualitative and mixed method research, HIV and AIDS.  Non-author data collectors were recruited based on their prior experience on qualitative research, and received ten-day long training on qualitative data collection and analysis, community and health system readiness, HIV and AIDS. |
| *Relationship with participants* | | | |
| Relationship established | 6 | Was a relationship established prior to study commencement? | No, no relationship established between researchers and research participants. |
|  | 7 | What did the participants know about the researcher? e.g. personal goals, reasons for doing the research | Nothing, except from reasons for conducting research, which was described to the research participants to receive written or verbal consent.  Page no. 12 |
| Interviewer characteristics | 8 | What characteristics were reported about the inter viewer/facilitator? e.g. Bias, assumptions, reasons and interests in the research topic | We do not believe that there were any biases significant to the study findings. The researchers were fully aware about the potential bias, had long experience of conducting qualitative research. All the researchers had ethics certification on conducting research on human subjects; and GG, MNMK, AMRH, GS, MMR, AKMMR and SIK were certified on Results-based management (RBM). Besides, the research team followed several mechanisms to minimize bias of qualitative data collection and analysis.  Page no. 14 |
| **Domain 2: Study design** | | | |
| *Theoretical framework* | | | |
| Methodological orientation and Theory | 9 | What methodological orientation was stated to underpin the study? e.g. grounded theory, discourse analysis, ethnography, phenomenology,  content analysis | In our study, we aimed to explore and understand the willingness of the key population (KP) communities to receive STI services from public health facilities. For analyzing qualitative data, we followed thematic and contextual analysis techniques.  Page no. 13 |
| *Participant selection* | | | |
| Sampling | 10 | How were participants selected? e.g. purposive, convenience,  consecutive, snowball | The participants were purposively selected following the maximum variation, intensity and critical case sampling procedures to explore diverse issues related to the study objectives.  Page no. 11 |
| Method of approach | 11 | How were participants approached? e.g. face-to-face, telephone, mail,  Email | Participants were selected though service centers, known as Drop-In Centers (DIC). In addition, research guides, who were from KP communities (i.e., MSM, female sex workers, *hijra* and PWID) worked with the researchers to approach the research participants. Face-to-face interviews were conducted.  Page no. 11 |
| Sample size | 12 | How many participants were in the study? | We conducted 34 in-depth interviews with KP communities. Besides, we conducted nine key informant interviews (KIIs) and 11 focus group discussions (FGDs) with KPs, community leaders and leaders of Community Based Organizations (CBOs).  Page no. 10-11 |
| Non-participation | 13 | How many people refused to participate or dropped out? Reasons? | No participant refused to participate. |
| *Setting* | | | |
| Setting of data collection | 14 | Where was the data collected? e.g. home, clinic, workplace | Data was collected at DICs where the KPs came to receive a range of STI, behavior change services, and health products. Data were also collected at spots -- geographical areas where KPs usually gather. If either of these setting was not convenient, data were collected at a setting suitable for the research participants. These settings included streets, open areas like bust terminals or front of hospitals, and home of the participants. |
| Presence of non-  participants | 15 | Was anyone else present besides the participants and researchers? | No, no non-participant was present besides the participants and researchers. |
| Description of sample | 16 | What are the important characteristics of the sample? e.g. demographic  data, date | Participants were selected through maximum variation sampling procedure. The socio-demographic characteristics of the participants are provided in details on Table 1.  Page no. 15-17 |
| *Data collection* | | | |
| Interview guide | 17 | Were questions, prompts, guides provided by the authors? Was it pilot tested? | Yes, semi structured interview and FGD guidelines were field tested by the researchers. In addition, considering the emerging nature of qualitative data, the research team interpreted and exchanged findings through peer-debriefing meetings, which helped us modify data collection guidelines as needed.  Page no. 11 |
| Repeat interviews | 18 | Were repeat inter views carried out? If yes, how many? | None. |
| Audio/visual recording | 19 | Did the research use audio or visual recording to collect the data? | Interviews and FGDs were recorded using tape recorders where the participants gave verbal consent to record the interviews. Recordings of interviews and FGDs were transcribed in verbatim. |
| Field notes | 20 | Were ﬁeld notes made during and/or after the interview or focus group? | The researchers used field notes while conducting the interviews. During FGDs, while one researcher facilitated the FGD, one researcher was resent to take notes on non-verbal communications and other important field notes. |
| Duration | 21 | What was the duration of the inter views or focus group? | Each interview took one hour to one and a half hours. The duration of FGDs varied from one and a half hours to two hours. |
| Data saturation | 22 | Was data saturation discussed? | Yes, data saturation was discussed during the research process. We stopped data collection at the point of data saturation and point data of redundancy. |
| Transcripts returned | 23 | Were transcripts returned to participants for comment and/or correction? | No. |
| **Domain 3: analysis and ﬁndings** | | | |
| *Data analysis* | | | |
| Number of data coders | 24 | How many data coders coded the data? | GG, MNMK and AHRH coded the data for this research project. |
| Description of the coding  tree | 25 | Did authors provide a description of the coding tree? | Structural coding method by developing joint or collaborative coding framework was followed. Coding tree was not discussed, but available upon request.  Page no. 13 |
| Derivation of themes | 26 | Were themes identiﬁed in advance or derived from the data? | Themes derived/emerged from the data.  Page no. 13, 18 |
| Software | 27 | What software, if applicable, was used to manage the data? | Qualitative data analysis software was not used to manage the data. |
| Participant checking | 28 | Did participants provide feedback on the ﬁndings? | Yes, participants check took place during peer debriefing sessions.  Page no. 14 |
| *Reporting* | | | |
| Quotations presented | 29 | Were participant quotations presented to illustrate the themes/ﬁndings?  Was each quotation identiﬁed? e.g. participant number | Yes.  Page no. 18-33 |
| Data and ﬁndings consistent | 30 | Was there consistency between the data presented and the ﬁndings? | Yes.  Page no. 18-33 |
| Clarity of major themes | 31 | Were major themes clearly presented in the ﬁndings? | Yes, presented in the findings.  Page no. 18-33 |
| Clarity of minor themes | 32 | Is there a description of diverse cases or discussion of minor themes? | Yes.  Page no. 18-33 |

**Developed from:** Tong A, Sainsbury P, Craig J. Consolidated criteria for reporting qualitative research (COREQ): a 32-item checklist for interviews and focus groups. *International Journal for Quality in Health Care*. 2007. Volume 19, Number 6: pp. 349 –357.

1. Tong A, Sainsbury P, Craig J. Consolidated criteria for reporting qualitative research (COREQ): a 32-item checklist for interviews and focus groups. International Journal for Quality in Health Care. 2007;19(6):349-57.
